# Craniofacial morphology and growth in Muenke syndrome, Saethre-Chotzen syndrome, and *TCF12*-related craniosynostosis

**DOI:** 10.1007/s00784-021-04275-y

**Published:** 2021-12-14

**Authors:** T. M. Choi, O. W. Lijten, I. M. J. Mathijssen, E. B. Wolvius, E. M. Ongkosuwito

**Affiliations:** 1 Erasmus MC, University Medical Center Rotterdam, Department of Oral Maxillofacial Surgery, Special Dental Care and Orthodontics, Dutch Craniofacial Center, Rotterdam, The Netherlands; 2 Erasmus MC, University Medical Center Rotterdam, Department of Plastic and Reconstructive Surgery and Hand Surgery, Dutch Craniofacial Center, Rotterdam, the Netherlands; 3 Department of Dentistry, Section of Orthodontics and Craniofacial biology, Radboud University Medical Center, Nijmegen, the Netherlands

**Keywords:** Midface hypoplasia, Jaw relationship, Syndromic craniosynostosis, Craniofacial anomalies, Growth/development, Orthodontic(s)

## Abstract

**Objectives:**

To determine whether the midface of patients with Muenke syndrome, Saethre-Chotzen syndrome, or *TCF12-*related craniosynostosis is hypoplastic compared to skeletal facial proportions of a Dutch control group.

**Material and methods:**

We included seventy-four patients (43 patients with Muenke syndrome, 22 patients with Saethre-Chotzen syndrome, and 9 patients with *TCF12*-related craniosynostosis) who were referred between 1990 and 2020 (age range 4.84 to 16.83 years) and were treated at the Department of Oral Maxillofacial Surgery, Special Dental Care and Orthodontics, Children’s Hospital Erasmus University Medical Center, Sophia, Rotterdam, the Netherlands. The control group consisted of 208 healthy children.

**Results:**

Cephalometric values comprising the midface were decreased in Muenke syndrome (ANB: β = –1.87, *p* = 0.001; and PC1: *p* < 0,001), Saethre-Chotzen syndrome (ANB: β = –1.76, *p* = 0.001; and PC1: *p* < 0.001), and *TCF12*-related craniosynostosis (ANB: β = –1.70, *p* = 0.015; and PC1: *p* < 0.033).

**Conclusions:**

In this study, we showed that the midface is hypoplastic in Muenke syndrome, Saethre-Chotzen syndrome, and *TCF12*-related craniosynostosis compared to the Dutch control group. Furthermore, the rotation of the maxilla and the typical craniofacial buildup is significantly different in these three craniosynostosis syndromes compared to the controls.

**Clinical relevance:**

The maxillary growth in patients with Muenke syndrome, Saethre-Chotzen syndrome, or *TCF12*-related craniosynostosis is impaired, leading to a deviant dental development. Therefore, timely orthodontic follow-up is recommended. In order to increase expertise and support treatment planning by medical and dental specialists for these patients, and also because of the specific differences between the syndromes, we recommend the management of patients with Muenke syndrome, Saethre-Chotzen syndrome, or *TCF12*-related craniosynostosis in specialized multidisciplinary teams.

**Supplementary Information:**

The online version contains supplementary material available at 10.1007/s00784-021-04275-y.

## Introduction

Midface hypoplasia is one of the clinical features that was reported in Muenke syndrome and Saethre-Chotzen syndrome [1–5]. In the past, Muenke syndrome, Saethre-Chotzen syndrome, and *TCF12*-related craniosynostosis were often undiagnosed or misdiagnosed because of the mild and sometimes overlapping clinical features [4, 6–9]. The main overlapping clinical feature of these three syndromes is coronal suture synostosis. The distinctive main features of Muenke syndrome are carpal and tarsal fusions and hearing loss [4]. Distinctive main features of Saethre-Chotzen syndrome are strabismus and ptosis [10]. Because the mutation that causes *TCF12*-related craniosynostosis is recently discovered [6], no distinctive main features of this syndrome has been reported to date. Additionally, the rarity of these three craniosynostosis syndromes makes it relatively difficult for unexperienced clinicians to correctly recognize or diagnose these syndromes. The prevalence of Muenke syndrome is 1:10,000–12,500 among newborns [2]. The prevalence of Saethre-Chotzen syndrome is 1:25,000–50,000 among newborns [11, 12]. The prevalence of *TCF12*-related craniosynostosis is not yet determined because this syndrome was recently discovered [6]. In order to prevent misdiagnosis of craniosynostosis patients, genetic testing of these patients and their parents is now widely and commonly applied. Genetic confirmation for Muenke syndrome, Saethre-Chotzen syndrome [2, 11, 12], and *TCF12*-related craniosynostosis [6] has been possible for several years. Yet, no extensive cephalometric study has been carried out, quantifying the midface deficiency in these three craniosynostosis syndromes. Furthermore, only one cephalometric study with limited cohort size indicated that patients with Muenke syndrome did not have midface hypoplasia compared to the individuals of the control group [14]. Therefore, it remains unclear whether or not midface hypoplasia is characteristic in Muenke syndrome, Saethre-Chotzen syndrome, and *TCF12*-related craniosynostosis and whether or not it is of clinical significance.

Midface hypoplasia is clearly a characteristic clinical feature in severe craniosynostosis syndromes such as Apert syndrome and Crouzon syndrome. Subsequently, in these syndromes, this results in deviant dental arch dimensions [15]. Smaller maxillary dental arch dimensions also have been found in Muenke syndrome, Saethre-Chotzen syndrome, and *TCF12*-related craniosynostosis [16]. Therefore, we expect that midface hypoplasia would also be present in these syndromic craniosynostosis patients. Furthermore, it is important to determine the severity of midface hypoplasia in these syndromes in order to determine the best timing of the start of orthodontic treatment and whether maxillofacial surgery is necessary in children with Muenke syndrome, Saethre-Chotzen syndrome, and *TCF12-*related craniosynostosis [17].

The aim of our study is to compare the skeletal, sagittal, and vertical cephalometric dimensions of patients with Muenke syndrome, Saethre-Chotzen syndrome, and *TCF12*-related craniosynostosis to the individuals of a control group of healthy Dutch children.

## Material and methods

This retrospective case–control study was approved by the Medical Ethics Committee of the Erasmus University Medical Centre in Rotterdam, the Netherlands (MEC-2013–536). Lateral cephalograms were part of orthodontic documentation required in the treatment protocol used by the craniofacial team in the Erasmus University Medical Centre Rotterdam, the Netherlands. For the analog lateral cephalograms, landmarks were drawn on tracing paper and digitized afterwards. On the digitized tracing paper and digital lateral cephalograms, digital measurements were made in Viewbox software (version 3.1.1.12; dHal orthodontic Software, Athens, Greece).

### Patient sample

**Fig. 1:**
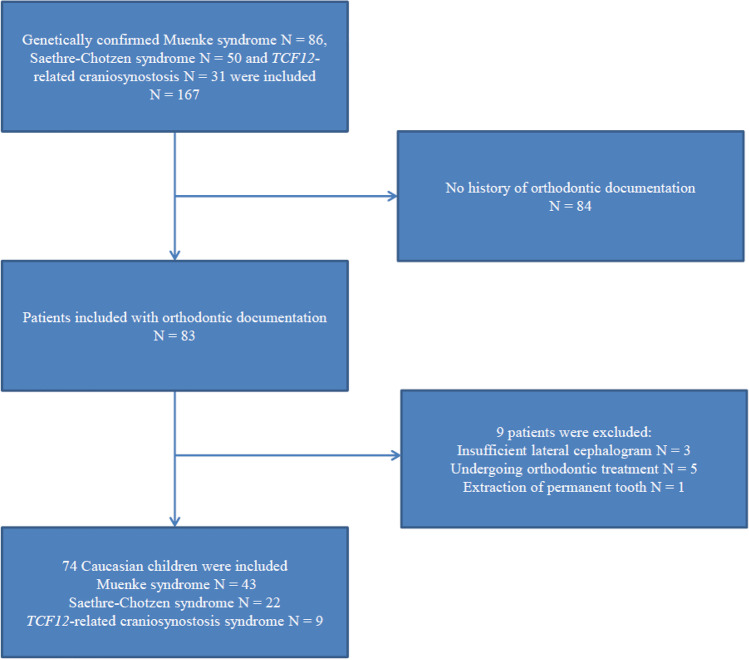
Flowchart displaying the inclusion and exclusion criteria of patients and the final study group

This study included 167 Caucasian children that were referred between 1990 and 2020 to the craniofacial team in Erasmus University Medical Centre Rotterdam, the Netherlands. The clinical diagnosis was determined by a craniofacial expert (e.g., a clinical geneticist and/or a plastic surgeon). In all patients, the diagnosis was confirmed molecularly. According to the craniofacial teams’ protocol, documentation with lateral cephalograms started at the age of 6 years and ended when the patient turned 18 years. We searched for available lateral cephalograms for all 167 patients (Muenke syndrome, *n* = 86; Saethre-Chotzen, *n* = 50; *TCF12*-related craniosynostosis, *n* = 31), and we selected lateral cephalograms of sufficient quality that were taken in natural head position and central occlusion. Patients were excluded when they had no documented lateral cephalograms, when the quality of the lateral cephalogram was insufficient, when they had extraction of teeth in the permanent dentition, when they had undergone any orthodontic treatment, or when they had undergone maxillary surgery (Fig. 1). Based on these criteria, we excluded 93 patients. Patients previously underwent one craniofacial surgical procedure (e.g., fronto-orbital advancement) according to the treatment protocol of the craniofacial team in the Erasmus University Medical Center Rotterdam, the Netherlands. None of the selected patients had a second craniofacial vault expansion. Only one patient never had craniofacial surgery.

The final study group (syndromic) consisted of 74 Dutch patients with a mean age of 9.46 years (SD 2.27) (43 females (mean age 9.58 (SD 2.20) and 31 males (mean age 9.25 (SD 2.39)). Of this sample, 43 patients had Muenke syndrome with a mean age of 9.82 (SD 2.22), 22 patients had Saethre-Chotzen syndrome with a mean age of 8.47 (SD 2.06), and 9 patients had *TCF12* with a mean age of 10.00 (SD 2.41).

The included lateral cephalograms were taken from patients between 4.84 and 16.83 years and who were born between 1982 and 2015.

### Control group

The control group consisted of 208 Caucasian children without any syndrome or congenital facial anomaly. The mean age was 9.39 years (SD 0.59), 102 were boys (mean age: 9.39 (SD 0.52)), and 106 were girls (mean age 9.39 (SD 0.66)). All patients were part of the population-based cohort study from the Nijmegen Growth Study (NGS), Nijmegen, the Netherlands [18]. The Nijmegen Growth Study was a mixed longitudinal, interdisciplinary study of growth and development of healthy Dutch children between 4 and 14 years old and was conducted between 1971 and 1976.

### Cephalometric measurements

We determined the following values in the cephalometric analysis for sagittal skeletal measurements such as SNA (sella-nasion-A point), SNB (sella-nasion-B point), and ANB (A point-nasion-B point); for vertical skeletal measurements such as NSL/ML (sella-nasion line/mandibular line), NL/ML (nasal line/mandibular line), and NSL/NL (sella-nasion line/nasal line); and for dental-basal/dental measurements such as Ils/NL (inclination of the upper incisors relative to the nasal line), interincisal angle, Ili/ML (inclination of the lower incisors relative to mandibular line), and NSL/BOP (rotation of the occlusal plane relative to the sella-nasion line) (Table 1).

**Table 1:** Description of cephalometric measurements

	Description of cephalometric measurements to determine the	Relative to	Landmarks
Sagittal skeletal			
SNA	Position of the maxilla	Cranial base	SNA
SNB	Position of the mandible	Cranial base	SNB
ANB	Jaw relationship	Nasion	ANB
Vertical skeletal			
NSL/ML	Growth direction of the skeletal pattern	Cranial base	SN line/Go-Me
NL/ML	Growth direction of the jaw complex		ANS-PNS/Go-Me
NSL/NL	Rotation direction of palatal plane	Cranial base	SN line/ANS-PNS
Dental-basal/dental			
Ils/NL	Angle of upper incisors	Palatal plane	
Interincisal angle	Angle of the incisors		
Ili/ML	Inclination of lower incisors	Mandibular plane	
NSL/BOP	Rotation of occlusal plane	Cranial base	SN line/tip of U1 and M1

### Measurement error

To determine intra-rater reliability, one rater rescored 20 randomly selected lateral cephalograms at 2 weeks after the first scores were determined. Inter-rater reliability was determined by having a second rater measure the same 20 lateral cephalograms. The intra-rater reliability and the inter-rater agreement were calculated with the intra-class correlation coefficient (ICC). A correlation coefficient of at least 0.75 was considered to indicate high reliability [19].

### Statistics

Continuous data are presented as mean ± standard deviation. Categorical data are presented as number and proportion. We used histograms to assess normality of data. Parametric tests were used for normally distributed data and nonparametric tests for non-normally distributed data. For statistical purposes, we analyzed the lateral cephalogram of the patient that was closest to the mean age of the control group.

Age was compared between the groups with Kruskal–Wallis test and sex with chi-square test. Because all the cephalometric measurements were normally distributed, we used ANOVA test to compare them between the three syndromes and the controls. We used Bonferroni correction to account for the comparison in 10 variables. Each variable that was significantly different among the groups was subsequently compared between each syndrome and controls using linear regression analysis in which we adjusted for age and sex. Outcomes of linear regression analysis are presented as unstandardized beta (*β*) with 95% confidence interval (CI) and *p* value.

Because several cephalometric measurements were strongly correlated with each other, we performed a principal component analysis as has been done previously by Halazonetis in 2004 [20]. The principal component analysis is described in detail in Supplementary information 1. For each principal component, a standardized component score was saved for each child, enabling us to compare PC scores between patients with Muenke syndrome, Saethre-Chotzen syndrome, *TCF12*-related craniosynostosis, and the controls. The principal component scores that were compared between Muenke syndrome, Saethre-Chotzen syndrome, *TCF12*-related craniosynostosis, and controls were adjusted for age and sex in the regression analysis. A *p* value below 0.05 was considered as statistically significant. Statistical analyses were performed with SPSS Statistics version 24.0 (IBM Corp. Armonk, NY, USA).

## Results

### Intra-class correlation coefficient

The ICC for intra-observer reliability was excellent for ANB (0.983), NSL/NL (0.912), and interincisal angle (0.914); good for SNA (0.820), SNB (0.804), Ili/ML (0.853), Ils/NL (0.856), and NSL/BOP (0.807); and moderate for NSL/ML (0.573) and NL/ML (0.743).

The ICC for inter-observer reliability was good for SNA (0.801), SNB (0.822), ANB (0.838), NSL/NL (0.851), interincisal angle (0.789), and moderate for NSL/ML (0.697), NL/ML (0.619), Ils/NL (0.618), Ili/ML (0.618) and NSL/BOP (0.725) (Table 2).

**Table 2:** Intra-observer and inter-observer reliability correlation coefficient for the different measurements

	Intra-observer reliability correlation coefficient	Inter-observer reliability correlation coefficient
SNA angle (deg)	0.820	0.801
SNB angle (deg)	0.804	0.822
ANB angle (deg)	0.983	0.838
NSL/ML angle (deg)	0.573	0.697
NL/ML angle (deg)	0.743	0.619
NSL/NL angle (deg)	0.912	0.851
Ils/NL angle (deg)	0.856	0.618
Interincisal angle (deg)	0.914	0.789
Ili/ML angle (deg)	0.853	0.618
NSL/BOP angle (deg)	0.807	0.725

### Study population

The study population consisted of 43 patients with Muenke syndrome, 22 with Saethre-Chotzen syndrome, 9 with *TCF12*-related craniosynostosis, and 208 controls. Baseline characteristics are shown in Table 3. Differences were found in age (*p* = 0.012) but not in sex (*p* = 0.568) between Muenke syndrome, Saethre-Chotzen syndrome, *TCF12*-related craniosynostosis, and the controls (Table 3).

**Table 3:** Baseline characteristics

	Muenke syndrome *N* = 43	SCS *N* = 22	*TCF12* *N* = 9	Controls *N* = 208	***p*** value*
Age	9.8 ± 2.2 (4.8–16.8)	8.4 ± 2.1 (5.8–14.9)	10.0 ± 2.4 (6.7–15.0)	9.4 ± 0.6 (4.1–11.5)	0.012
Female, *n* (%)	23 (53.5%)	14 (63.6%)	6 (66.7%)	106 (51.0%)	0.568

Data are presented as mean ± standard deviation unless otherwise specified. *Kruskal–Wallis test for age and chi-square test for sex. SCS = Saethre-Chotzen syndrome. *TCF12* = *TCF12*-related craniosynostosis. The age range of the group is displayed below the mean age between parentheses

#### Comparison of individual cephalometric measurements

The comparison of the cephalometric measurements is presented in Table 4. After Bonferroni correction of the outcomes with ANOVA analysis, we found that SNB, ANB, NSL/NL, SN/ML, NL/ML, and NSL/BOP differed statistically significant between patients with Muenke syndrome, Saethre-Chotzen syndrome, *TCF12*-related craniosynostosis, and the controls. Cephalometric measurements that were significantly different in the ANOVA analysis were adjusted for age and sex and compared between the three syndromes and the control group (Table 5).

**Table 4:** Comparison of cephalometric measurements between the three syndromes and controls

	Muenke syndrome *N* = 43	SCS *N* = 22	*TCF12* *N* = 9	Controls *N* = 208	*p* value*
SNA	80.41 ± 4.49	76.89 ± 6.10	81.10 ± 3.36	79.98 ± 3.33	1.000
SNB	78.18 ± 4.96	74.00 ± 5.06	78.62 ± 3.30	75.60 ± 3.22	< 0.001
ANB	2.25 ± 3.58	2.90 ± 3.56	2.48 ± 3.26	4.40 ± 1.94	< 0.001
NSL/NL	1.80 ± 3.83	12.78 ± 4.40	5.90 ± 3.88	8.42 ± 3.15	< 0.001
SN/ML	36.03 ± 6.74	45.31 ± 9.90	43.19 ± 3.86	36.77 ± 4.71	< 0.001
NL/ML	34.23 ± 5.23	32.53 ± 8.81	37.28 ± 6.34	28.35 ± 4.96	< 0.001
Ils/NL	106.14 ± 7.73	108.19 ± 9.19	106.92 ± 6.13	108.04 ± 9.16	1.000
Interincisal angle	132.46 ± 10.22	132.54 ± 12.22	132.98 ± 6.70	129.25 ± 12.85	1.000
Ili/ML	89.14 ± 6.60	89.61 ± 10.43	85.62 ± 3.51	87.76 ± 7.01	1.000
NSL/BOP	10.41 ± 5.76	21.09 ± 6.83	13.46 ± 4.49	18.72 ± 4.11	< 0.001

Data are presented as mean ± standard deviation. ANOVA test was used to compare the cephalometric measurements between groups. *p value after Bonferroni correction for multiple testing with 10 variables

**Table 5:** Comparison of cephalometric measurements between each syndrome and controls adjusted for age and sex

	Muenke syndrome			SCS			*TCF12*		
	*β*	CI	*p* value	*β*	CI	*p* value	*β*	CI	*p* value
SNB	2.25	[1.09, 3.41]	< 0.001	–1.63	[–3.23, –0.03]	0.046	2.87	[0.67, 5.08]	0.011
ANB	–1.87	[–2.60, –1.13]	< 0.001	–1.76	[–2.76, –0.77]	0.001	–1.70	[–3.06, –0.34]	0.015
NSL/NL	–6.46	[–7.55, –5.37]	< 0.001	4.86	[3.34, 6.38]	< 0.001	–2.48	[–4.65, –0.30]	0.026
SN/ML	–0.46	[–2.15, 1.24]	0.597	8.66	[6.14, 11.18]	< 0.001	6.32	[3.13, 9.51]	< 0.001
NL/ML	6.01	[4.33, 7.68]	< 0.001	3.80	[1.26, 6.33]	0.004	8.78	[5.36, 12.21]	< 0.001
NSL/BOP	–7.51	[–9.05, –5.96]	< 0.001	2.27	[0.06, 4.47]	0.044	–5.02	[–7.83, –2.20]	0.001

Data are presented as unstandardized beta, 95% confidence interval, and *p* value

After adjusting for age and sex and Bonferroni correction for multiple testing, we found that patients with Muenke syndrome had an increased SNB (*p* = 0.001), decreased ANB (*p* < 0.001), decreased NSL/NL (*p* < 0.001), increased NL/ML (*p* < 0.001), and decreased NSL/BOP (*p* < 0.001) compared to the controls. Patients with Saethre-Chotzen syndrome had an increased SNA (*p* < 0.001), decreased SNB (*p* = 0.046), decreased ANB (*p* = 0.001), increased NSL/NL (*p* < 0.001), increased SN/ML (*p* < 0.001), increased NL/ML (*p* = 0.004), and increased NSL/BOP (*p* = 0.044) compared to the controls. Lastly, patients with *TCF12-*related craniosynostosis had an increased SNB (*p* = 0.011), decreased ANB (*p* = 0.015), decreased NSL/NL (*p* = 0.026), increased SN/ML (*p* < 0.001), increased NL/ML (*p* = 0.001), and decreased NSL/BOP (*p* = 0.001) compared to controls (Table 5).

**Fig. 2:**
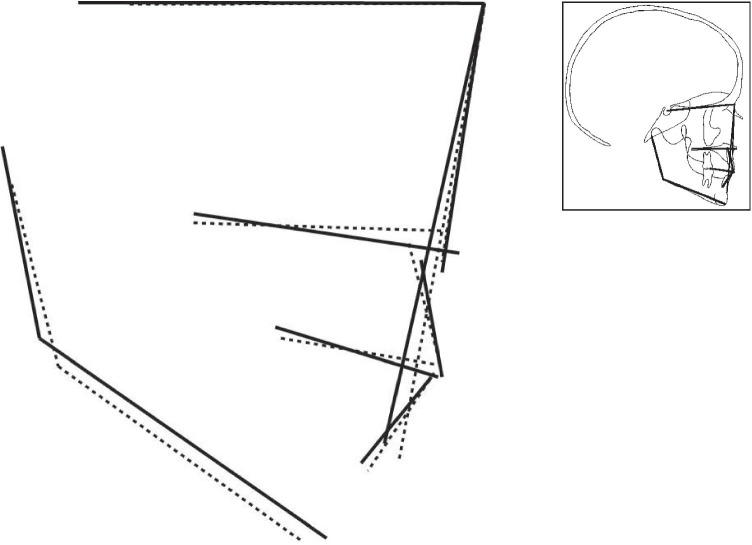
Visualization of the cephalometric profile of patients with Muenke syndrome (dashed line) versus the control group (black line) by superposition on the cranial base, based on the calculated average cephalometric values for each group

**Fig. 3:**
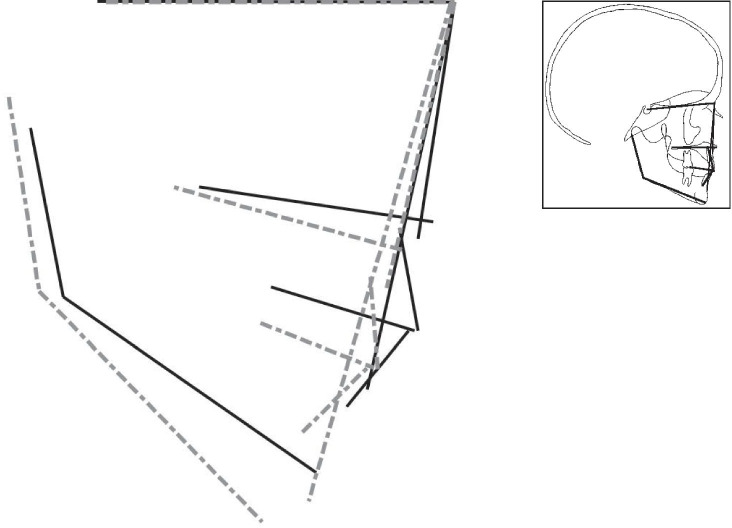
Visualization of the cephalometric profile of patients with Saethre-Chotzen syndrome (dashed line) versus the control group (black line) by superposition on the cranial base, based on the calculated average cephalometric values for each group

**Fig. 4:**
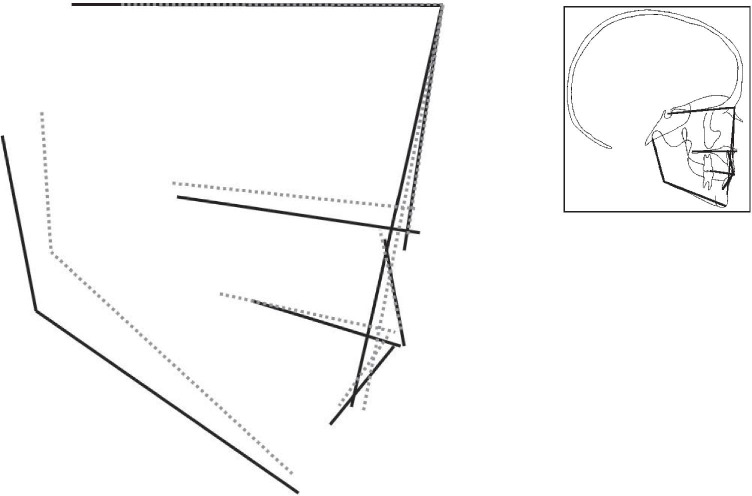
Visualization of the cephalometric profile of patients with *TCF12*-related craniosynostosis (dashed line) versus the control group (black line) by superposition on the cranial base, based on the calculated average cephalometric values for each group

Figures 2 to 4 display the superimposition of the average cephalometric values of each syndromic patient compared to the average cephalometric values of the control patient. The superimposition is made on the cranial base and point nasion. The tracings are constructed from the outcome of the mean values for each cephalometric variable as shown in Table 4 with the correct magnification factor.

#### Principal component analysis

We included 4 principal components with an eigenvalue above 1, which explained 78.9% of the total variance. PC1 consisted of SNA, SNB, NSL/NL, SN/ML, and NSL/BOP. PC2 consisted of NSL/NL, NL/ML, and Ils/NL. PC3 consisted of NL/ML and interincisal angle. PC4 consisted of SNA, ANB, and Ils/NL. Supplementary information 1 shows a detailed description of the principal component analysis procedure.

We then compared the 4 principal components between the three syndromes and controls. Patients with Muenke syndrome had decreased values of PC1, PC2, PC3, and PC4 compared to the controls, adjusted for age and sex. Patients with Saethre-Chotzen syndrome had increased PC1 and PC3 and decreased PC2 and PC4 compared to the controls, adjusted for age and sex. Lastly, patients with *TCF12-*related craniosynostosis had decreased PC1, PC2, PC3, and PC4 compared to the controls, adjusted for age and sex (Table 6).

**Table 6:** Comparison of PC between each syndrome and controls, adjusted for age and sex

	Muenke syndrome			SCS			*TCF12*		
	*β*	CI	*p* value	*β*	CI	*p* value	*β*	CI	*p* value
PC1	–1.05	[–1.35, –0.75]	< 0.001	0.87	[0.45, 1.28]	< 0.001	–0.56	[–1.08, –0.04]	0.033
PC2	–1.03	[–1.35, –0.72]	< 0.001	–0.42	[–0.89, 0.04]	0.075	–1.27	[–1.88, –0.67]	< 0.001
PC3	–0.36	[–0.73, 0.00]	0.051	0.20	[–0.32, 0.72]	0.447	–0.18	[–0.89, 0.52]	0.608
PC4	–0.76	[–1.06, –0.46]	< 0.001	–0.68	[–1.12, –0.24]	0.003	–0.54	[–1.09, 0.01]	0.052

PC1 consisted of SNA, SNB, NSL/NL, SN/ML, and NSL/BOP. PC2 consisted of NSL/NL, NL/ML, and Ils/NL. PC3 consisted of NL/ML and interincisal angle. PC4 consisted of SNA, ANB, and Ils/NL

## Discussion

This retrospective case–control study indicates that children with Muenke syndrome, Saethre-Chotzen syndrome, and *TCF12*-related craniosynostosis have distinctive skeletal and dental characteristics. The midface is hypoplastic in Muenke syndrome, Saethre-Chotzen syndrome, and *TCF12*-related craniosynostosis compared to those of the Dutch controls. In our visual presentation of Muenke syndrome, the hypoplastic midface is also represented by an increased value of the mandible (SNB) and a decreased value of the jaw relationship (ANB) (Fig. 2). One other cephalometric study also shows a significant smaller value of SNA in their patients, similar to our outcome in Muenke syndrome [14]. Various other case studies have reported their clinical observation of midface hypoplasia in Muenke syndrome [2, 5, 21]. To our best knowledge, this is the first cephalometric cohort study that shows that the midface is hypoplastic in Muenke syndrome. The superimposition of a patient with Muenke syndrome displays that the nasal floor is more anteriorly rotated. Therefore, the skeletal pattern is concealed and seems to be not different from the Dutch controls. Additionally, the jaw complex is more hyperdivergent compared to the Dutch controls (Fig. 2). Premature fusion of circummaxillary sutures may play a role in affecting the vertical maxillary growth negatively in patients with Muenke syndrome. This is displayed by a decreased value of NSL/NL, which means a more anteriorly rotation of the maxilla [22–24].

In our visual presentation of Saethre-Chotzen syndrome (Fig. 3), the maxilla (SNA) is retruded, and the jaw relationship (ANB) has a decreased value compared to the Dutch controls. The midface is hypoplastic in Saethre-Chotzen syndrome compared to the Dutch controls. In contrast to Muenke syndrome, the vertical maxillary growth is not decreased compared to the Dutch controls. The maxilla in Saethre-Chotzen syndrome displays a more posterior rotation compared to the controls. Surprisingly, this is a different vertical growth pattern of the maxilla in Saethre-Chotzen syndrome compared to the Muenke syndrome. In Muenke syndrome, the pattern seems to have a posterior vertical inhibition, while Saethre-Chotzen syndrome tends to have an anterior vertical inhibition. The difference in genes causing craniosynostosis and the timing of fusion of circummaxillary sutures may play a role in causing a different growth pattern of the midface in these two syndromes [22–24]. Additionally, the skeletal pattern and jaw complex are more hyperdivergent in Saethre-Chotzen syndrome compared to the Dutch controls. The more hyperdivergent jaw complex is also seen in Muenke syndrome and *TCF12*-related craniosynostosis. This may be a distinctive skeletal growth pattern in these three coronal craniosynostosis syndromes (Figs. 2–4).

In our visual presentation of *TCF12*-related craniosynostosis, the mandible (SNB) has an increased value, and the jaw relationship (ANB) has a decreased value. The vertical maxillary growth pattern is decreased by a more anteriorly rotated nasal floor compared to the controls. This finding corresponds to the vertical maxillary growth pattern in Muenke syndrome. The skeletal pattern and jaw complex in *TCF12*-related craniosynostosis are more hyperdivergent compared to the Dutch controls. These results correspond to the skeletal pattern and jaw complex that we have found in Saethre-Chotzen syndrome. Previously reported shared clinical features of Saethre-Chotzen syndrome and *TCF12*-related craniosynostosis are now supported by our cephalometric findings [4, 6–9] (Figs. 3 and 4).

The skeletal jaw relationship and facial divergence, which are characterized by SNA, SNB, NSL/NL, SN/ML, and NSL/BOP, showed that children with Muenke syndrome and *TCF12*-related craniosynostosis have a decreased PC1, indicating that sella is located vertically lower compared to patients of the Dutch control group. SNB has an increased value in Muenke syndrome and *TCF12*-related craniosynostosis, which can be explained by significantly more counterclockwise rotation of the mandible that is the result of a more anterior rotation of the palatal plane angle. PC1 analysis in the Saethre-Chotzen syndrome was increased and showing a posterior rotation of palatal plane compared to control Dutch patients. The posterior rotation of the palatal plane results in clockwise rotation of the mandible, which, in contrast to Muenke and *TCF12*-related craniosynostosis, was not different compared to patients of the Dutch control group. The skeletal pattern which is not different in Muenke syndrome may be the result of a more counterclockwise rotation of the mandible.

The vertical growth pattern, jaw complex, and proclination of the upper incisors (PC2), which is characterized by NSL/NL, NL/ML, and Ils/NL, were decreased in the Muenke syndrome and *TCF12*-related craniosynostosis compared to patients of the Dutch control group. The proclination of the upper incisors can be the result of a relatively smaller maxilla in sagittal dimension with a normal mandible, causing the maxillary upper incisors to procline [16]. PC2 analysis for the Saethre-Chotzen syndrome was not different compared to patients of the Dutch control group.

The jaw complex and dental relationship (PC3), which is characterized by NL/ML and interincisal angle, were not different in Muenke syndrome, Saethre-Chotzen syndrome, and *TCF12*-related craniosynostosis compared to the Dutch controls.

This study had some limitations. Craniofacial malformations have a three-dimensional representation that is only partly covered by a lateral cephalogram, and therefore, it may be more difficult to record subtle changes compared to the Dutch population [25, 26]. Lateral cephalograms provide a lower radiation dose to patients compared to cone beam CTs and are also in use for a much longer period [27]. This provided us the data to report on a rare group of patients. Another potential limitation was that the children in our syndromic group were born almost two decennia later than the children in our Dutch control group. During that period, there was a positive temporal trend in body length among Dutch children [28]. We used angles instead of lengths to counter this problem. We aimed for homogeneous groups that included every patient available to us, but we are not sure whether the ratio of male/female patients is representative. Further research should aim towards a three-dimensional representation of these patients and when possible include further patients.

The overall results of our measurements are presented in the visual presentations of Muenke syndrome, Saethre-Chotzen syndrome, and *TCF12-*related craniosynostosis. These figures show the craniofacial buildup of the syndromes that are superimposed on those of the Dutch controls. Although the sagittal jaw relationship (ANB) in these three syndromes is similarly deviant, the overall cephalometric configuration of the craniofacial buildup shows considerable differences between the syndromes.

Patients with Muenke syndrome, Saethre-Chotzen syndrome, and *TCF12*-related craniosynostosis have a more vertical craniofacial buildup compared to the control group. Also, these syndromic patients have smaller dental arch dimensions [16]. Although these patients have distinctive craniofacial and dental features, the standard typical conventional therapy does not exist. No relationship between our cephalometric results and functional anomalies is known. The more vertical craniofacial buildup of these syndromic patients suggests that the retention protocol regarding stability after orthodontic treatment is even more important compared to patients with an average vertical craniofacial buildup. Further research is needed to determine whether a specific retention protocol is necessary in order to achieve the same results in stability after successful orthodontic treatment in patients with Muenke syndrome, Saethre-Chotzen syndrome, and *TCF12*-related craniosynostosis.

## Conclusion

In this study, we showed that the midface is hypoplastic in Muenke syndrome, Saethre-Chotzen syndrome, and *TCF12*-related craniosynostosis compared to the Dutch control group. Furthermore, the rotation of the maxilla and the typical craniofacial buildup is significantly different in these three coronal craniosynostosis syndromes compared to the Dutch population. When treating these patients, clinicians should include these results in their planning towards a harmonious profile.

## Supplementary Information

Below is the link to the electronic supplementary material.

Supplementary file1 (PDF 79 KB)
