# Supplementary material for: Craniofacial morphology and growth in Muenke syndrome, Saethre-Chotzen syndrome, and TCF12-related craniosynostosis
Source: Clin Oral Investig. 2021 Dec 14;26(3):2927–36. doi: 10.1007/s00784-021-04275-y (PMC8898243; doi:10.1007/s00784-021-04275-y)
Supplement: Supplementary file 1 — Supplementary file1 (PDF 79 KB) [file 784_2021_4275_MOESM1_ESM.pdf]

Title: Craniofacial morphology and growth in Muenke syndrome, Saethre-Chotzen syndrome and *TCF12*-related craniosynostosis

Journal name: Clinical Oral Investigations

Authors: T.M. Choi [1] DDS, O.W. Lijten [3] DDS, E.B. Wolvius [1] DDS, MD, PhD, I.M.J. Mathijssen [2] MD, PhD, E.M. Ongkosuwito [1,3] DDS, PhD.

1 Erasmus MC, University Medical Center Rotterdam, Department of Oral Maxillofacial Surgery, Special Dental Care and Orthodontics, Dutch Craniofacial Center, The Netherlands.

2 Erasmus MC, University Medical Center Rotterdam, Department of Plastic and Reconstructive Surgery and Hand Surgery, Dutch Craniofacial Center, the Netherlands.

3 Department of Dentistry, Section of Orthodontics and Craniofacial biology, Radboud University Medical Center, the Netherlands.

Address correspondence to:

T.M. Choi

Erasmus MC-Sophia

Secretariaat Orthodontie

Wytemaweg 80

3015 CN Rotterdam

the Netherlands

Telephone number: +31107036471

Fax number: +31107036810

E-mail t.choi@erasmusmc.nl

Supplement 1 Methods for principal component analysis

First, a correlation matrix was made for the 10 cephalometric measurements. The correlation matrix is shown in supplemental Table 1. Second, we selected the number of components based on the scree plot, Pearson correlation coefficient matrix and the eigenvalue of each principal component. All together, we included four principal components with each an eigenvalue above 1 and total explained variance of 78.9%. After comparing several rotation procedures, we applied a direct oblimin rotation. All cephalometric measurements, except Ili/ML had a primary loading above 0.5 (Supplement Table 2). Each measured cephalometric landmark with a primary loading below 0.5 or above 0.5 was included in the four principal components (Supplement Table 2). Therefore, SNA, SNB, NSL/NL, SN/ML and NSL/BOP were included in PC1. NSL/NL, NL/ML, and Ils/NL were included in PC2. NL/ML and interincisal angle were included in PC3. SNA, ANB and Ils/NL were included in PC4. Lastly, for each principal component a standardized component score was saved for each child, enabling us to compare PC scores between patients with Muenke syndrome, Saethre-Chotzen syndrome, *TCF12*-related craniosynostosis and the controls.

Supplement Table 1 Pearson correlation matrix for cephalometric variables

	SNA	SNB	ANB	NSL/NL	NSL/ML	NL/ML	Ils/NL	Interincisal.Angle	Ili / ML	NSL/BOP
SNA	1.00									
SNB	0.78	1.00								
ANB	0.32	-0.35	1.00							
NSL/NL	-0.41	-0.55	0.21	1.00						
NSL/ML	-0.52	-0.63	0.18	0.37	1.00					
NL/ML	-0.22	-0.23	0.02	-0.36	0.73	1.00				
Ils/NL	0.08	0.11	-0.05	0.15	-0.04	-0.15	1.00			
Interincisal angle	-0.12	-0.02	-0.16	0.05	-0.05	-0.09	-0.45	1.00		
Ili/ML	-0.01	0.01	-0.03	0.00	0.13	0.13	-0.13	0.05	1.00	
NSL/BOP	-0.48	-0.72	0.36	0.65	0.63	0.15	-0.06	-0.05	-0.01	1.00

Supplement Table 2 Factor loadings and communalities with a direct oblimin rotation applied

	PC1	PC2	PC3	PC4	Extraction
SNA	**-0.74**	0.18	0.30	**0.50**	0.93
SNB	**-0.92**	-0.06	0.08	-0.03	0.85
ANB	0.28	0.37	0.34	**0.78**	0.94
NSL/NL	**0.64**	**0.58**	-0.32	-0.03	0.85
NSL/ML	**0.84**	-0.30	0.31	-0.04	0.89
NL/ML	0.37	**-0.73**	**0.54**	-0.02	0.96

Ils/NL	-0.09	**0.54**	0.45	**-0.53**	0.78
Interincisal angle	0.01	-0.34	**-0.77**	0.22	0.75
Ili/ML	0.06	-0.33	0.01	0.19	0.15
NSL/BOP	**0.86**	0.23	-0.01	0.14	0.81
Explained variability	34.7%	16.9%	14.8%	12.5%	
All cephalometric variables with a factor loading below -0.5 or above 0.5 were included in the principal components and are depicted in bold.					
